# Optimising use of 4D-CT phase information for radiomics analysis in lung cancer patients treated with stereotactic body radiotherapy

**DOI:** 10.1088/1361-6560/abfa34

**Published:** 2021-05-24

**Authors:** Angela Davey, Marcel van Herk, Corinne Faivre-Finn, Sean Brown, Alan McWilliam

**Affiliations:** 1 Division of Cancer Sciences, School of Medical Sciences, Faculty of Biology, Medicine and Health, The University of Manchester, Manchester, United Kingdom; 2 Department of Radiotherapy Related Research, The Christie NHS Foundation Trust, Manchester, United Kingdom; 3 Department of Clinical Oncology, The Christie NHS Foundation Trust, Manchester, United Kingdom

**Keywords:** radiomics, 4D-CT, lung cancer, SABR, personalised, biomarkers

## Abstract

*Purpose*. 4D-CT is routine imaging for lung cancer patients treated with stereotactic body radiotherapy. No studies have investigated optimal 4D phase selection for radiomics. We aim to determine how phase data should be used to identify prognostic biomarkers for distant failure, and test whether stability assessment is required. A phase selection approach will be developed to aid studies with different 4D protocols and account for patient differences. *Methods*. 186 features were extracted from the tumour and peritumour on all phases for 258 patients. Feature values were selected from phase features using four methods: (A) mean across phases, (B) median across phases, (C) 50% phase, and (D) the most stable phase (closest in value to two neighbours), coined personalised selection. Four levels of stability assessment were also analysed, with inclusion of: (1) all features, (2) stable features across all phases, (3) stable features across phase and neighbour phases, and (4) features averaged over neighbour phases. Clinical-radiomics models were built for twelve combinations of feature type and assessment method. Model performance was assessed by concordance index (c-index) and fraction of new information from radiomic features. *Results*. The most stable phase spanned the whole range but was most often near exhale. All radiomic signatures provided new information for distant failure prediction. The personalised model had the highest c-index (0.77), and 58% of new information was provided by radiomic features when no stability assessment was performed. *Conclusion*. The most stable phase varies per-patient and selecting this improves model performance compared to standard methods. We advise the single most stable phase should be determined by minimising feature differences to neighbour phases. Stability assessment over all phases decreases performance by excessively removing features. Instead, averaging of neighbour phases should be used when stability is of concern. The models suggest that higher peritumoural intensity predicts distant failure.

## 1. Introduction

Stereotactic ablative radiotherapy (SABR) offers improved disease control and reduced toxicity compared to conventional radiotherapy for patients with early stage non-small cell lung cancer (NSCLC) (Nyman *et al* 2016). Despite success, around 20% of patients will experience distant failure within five years, and prognostic factors are not well developed (Senthi *et al* 2012, Loganadane *et al* 2016). Imaging biomarkers would be beneficial in this area for personalised treatment, or stratification in clinical trials, for example, to select patients that would benefit from surgery rather than SABR.

Radiomics, the extraction and analysis of quantitative features from medical images, could aid prognosis (Lambin *et al* 2012). For this patient group, most radiomic studies have been underpowered with sample sizes of approximately 100 patients or less (Huynh *et al* 2016, 2017, Li *et al* 2017, Oikonomou *et al* 2018, Lafata *et al* 2019), and few analyse four-dimensional computed tomography (4D-CT) data, which is used in lung cancer radiotherapy planning. As small tumours can exhibit large motion during respiration (Henry *et al* 2012), radiomic studies of free-breathing CT (FB-CT) are affected by motion variability (Fave *et al* 2015, Du *et al* 2019). The inclusion of 4D-CT could help overcome such issues, as each phase in the respiratory cycle displays a snapshot in time. With typically ten phases available (0%–90%), and composite reconstructions created for planning (such as the average intensity projection (AIP)), uncertainty remains in how to use this data in radiomic studies.

The simplest choice is the AIP reconstruction and tumour features (TFs) from AIP have out-performed FB-CT features for prediction of distant failure (Huynh *et al* 2017). However, the AIP represents average patient anatomy resulting in a blurred representation of a moving tumour. Instead, a single 4D phase is advised (Fornacon-Wood *et al* 2020), and end-exhale phase (50%) has out-performed both FB-CT and AIP for prediction of tumour histology (Lafata *et al* 2018). Commonly, AIP and end-exhale are mixed in analysis dependent on availability (Li *et al* 2017), despite the significant difference in feature values from each (Fave *et al* 2015). A single phase is useful to avoid a blurred representation of anatomy; however, the influence that different phase selection algorithms have on prognostic models is unknown.

End-exhale phase is often assumed the most stable for all patients (George *et al* 2006), but, there can be motion artifacts on any phase due to erratic breathing and heart motion (Fredberg Persson *et al* 2011). In addition, it is often assumed 50% is end-exhale, but this depends on how data is sorted. For phase angle sorting from peak inhale (standard in Philips scanners) the phase corresponding to end-exhale is not consistent across patients, as time spent in exhalation varies. Furthermore, the inspiration phase has been used in analysis without comparison (Franceschini *et al* 2020). A single pre-determined phase for all patients is potentially undesirable due to difference in data-binning implemented across institutions and individual patient differences. Instead, a phase selection tool to inform which phase is most suitable for each individual patient would account for different 4D protocols, and random patient and organ motion. Such an approach would aid multi-institutional studies.

So far, rather than investigating the prognostic value of different phase features, studies have sought features that agree across phases to reduce noise. Larue *et al* considered all phases (Larue *et al* 2017), whereas, Tanaka *et al* assessed phases neighbouring end-exhale (Tanaka *et al* 2019). Stability assessment will remove features descriptive of anatomical details that are sensitive to distortion (Larue *et al* 2017). This is of concern for distant failure prediction, as details from the tumour periphery can be linked to prognosis (Shimada *et al* 2010, Kadota *et al* 2015). Averaging feature values is another form of noise reduction (Zwanenburg *et al* 2019a), but the influence this has on prediction is unknown.

In this study, we aim to offer a data-driven approach to phase selection and study how to combine 4D phase information to offer best prognostic value. To do this, we compare four methods of obtaining a single feature set from phase feature values: (A) mean across all phases, (B) median across all phases, (C) 50% phase, or (D) selection of most stable phase for each patient based on similarity to neighbouring phases, coined the personalised approach. To fully analyse the 4D radiomics framework and make recommendations, we also compare methods of stability assessment by including: (1) all features, (2) features stable across 10 phases, (3) features stable across neighbour phases, and (4) all features but averaged across neighbour phases. 12 models will be created from suitable combinations of feature type and assessment method. This analysis will be performed in the clinical setting of distant failure prediction in early-stage NSCLC, as this is an area of unmet need that would benefit from an imaging biomarker to guide optimal management.

## 2. Material and methods

### 2.1. Data collection

273 stage I and IIa NSCLC patients (confirmed histologically or suspected based on radiology) were identified from patients treated with SABR for primary lung cancer during 2011–2017, in a single centre. Image, planning, and follow-up data were available. All patients had a motion-adapted gross tumour volume (iGTV) contoured in clinical practice, and were treated with 54 Gy in 3 fractions, or 60 Gy in 5 or 8 fractions.

4D-CT scans were acquired pre-treatment with the imaging protocol described in supplementary material (SM), section 1 (available online at stacks.iop.org/PMB/66/115012/mmedia) . 4D data was sorted into ten respiratory bins of equal time 0%–90%, where 0% phase represents peak inhale. All scans were reconstructed with slice thickness 3mm, and majority with pixel size 1.17 mm (range: 0.98–1.37 mm). Patients with missing image or treatment data, or complex treatment cases (i.e. more than one lesion) were removed from further analysis. Approval was granted to collect and analyse this patient data (REC reference: 17/NW/0060).

### 2.2. Patient follow-up

In agreement with UK guidelines (SABR UK Consortium 2019), patients underwent clinical follow-up four to six weeks after treatment. Patients were then followed-up every three months for the first year, and six monthly thereafter. A FB-CT was performed at the discretion of each clinician, with an 18F-FDG positron emission tomography (PET) scan and/or biopsy recommended when recurrence was suspected. For this study, follow-up data was collected retrospectively by a clinical team. Distant failure was defined as recurrence in an uninvolved lobe, contralateral lung, or any other extra-thoracic location. Time to distant failure was recorded from start of radiotherapy treatment to date of first scan that shown progression. If there was no recurrence, patients were censored at most recent follow-up.

Clinical variables available were tumour lobe location, T stage, age, sex, ECOG performance status (a grading 0–5 based on functional ability), ACE-27 comorbidity score (a grading mild–severe based on presence and severity of pre-existing medical conditions), and histological sub-type. Only patients in whom the clinical variable data collection was complete were considered in analysis against outcome.

### 2.3. Region-of-interest segmentation

**Figure 1.**
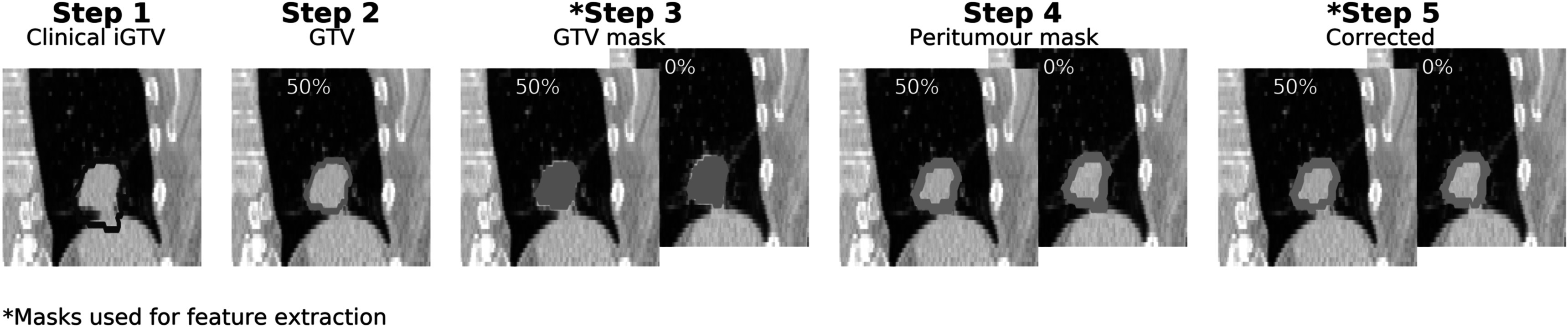
The steps implemented to obtain the regions-of-interest. Step 1 is the clinical iGTV. Step 2 shows the GTV contour generated on the 50% phase using an in-house method. Step 3 shows the contour sampled as a mask on phases, with 0% and 50% shown for an example, and Step 4 shows the peritumoural border sampled as a mask on the same phases. Step 5 shows the peritumoural border following correction for high-density normal tissue.

An in-house, validated method was implemented to generate a GTV on a reference phase (50%) from the iGTV for all patients (Davey *et al* 2020a). Briefly, local-rigid registration was used to obtain the translation set required to match the tumour position on each phase to the reference i.e. estimating tumour motion. The iGTV represents the volume mapped by the GTV over the motion trajectory, so from the iGTV and estimated motion the GTV can be derived (Step 2, figure 1). The GTV was mapped into the tumour position on every phase using the registration translation and masks were sampled (Step 3, figure 1).

Patients were excluded from analysis if classed as failed registration on visual assessment. Tumour motion amplitude was recorded by combining the difference in maximum and minimum positions from translation in all directions as a vector. Tumour volume was sampled from the 50% phase mask. Patients were excluded from analysis if tumour volume was below 64 voxels. This limit has been implemented in radiomics software (Nioche *et al* 2018), described as the volume below which texture features are meaningless.

A peritumoral border was sampled on every phase which was defined as a region 3mm inside and outside the GTV contour, following a published definition (Dou *et al* 2018). A correction algorithm was developed (SM, section 2) and applied to each phase to remove any high-density normal tissue (i.e. diaphragm, chest wall, or bone). After correction, only voxels which exist fully inside the corrected peritumoural border were included in analysis. The number of included voxels may differ across phases due to motion relative to chest wall and diaphragm position, or a smaller than voxel size motion amplitude. This would be the only reason for a change in volume across phases, as we assume there is no deformation of the tumour over the respiratory cycle. This assumption is based on biomechanics—as the tumour is a relatively rigid mass embedded in much softer lung tissue.

### 2.4. Feature extraction

An open source software, Pyradiomics version 2.2.0, was used in Python 3.6.9 for feature extraction (van Griethuysen *et al* 2017). Majority of features in this software are compliant with the Image Biomarker Standardisation Initiative (Zwanenburg *et al* 2019b, Fornacon-Wood *et al* 2020). 93 first order and texture features available were extracted with default settings, which comprised of feature groups: first order, symmetrical grey level co-occurrence matrix (GLCM), grey level size zone matrix, grey level run length matrix, neighbouring grey tone difference matrix, and grey level dependence matrix (GLDM). Features were extracted from both an original and filtered image, using a Laplacian of Gaussian (LoG) filter with sigma, *σ*, as 1.5 mm, to look at medium edge textures (Ganeshan *et al* 2010). Features were extracted from both the tumour and peritumoural regions-of-interest (ROIs), labelled TFs and peritumoural features (PFs) respectively, resulting in 372 features per phase.

Feature extraction was performed on the original voxel spacing without resampling, as almost 80% of images are the same pixel spacing, and all approximately 1mm with slice thickness 3 mm. Although isotropic resampling has been recommended, the best practice for implementation is an area of active research with no favoured approach (Van Timmeren *et al* 2020). A fixed bin size approach was implemented with bin-size of 25 Hounsfield Units (HU), the default setting in Pyradiomics. The lower bound on this data was −1024HU from the whole CT range, but varied for each patient as minimum value in the ROI. A re-segmentation lower bound was not defined as presence of air is potentially informative.

### 2.5. Creation of single feature set

Four methods (A)–(D) were implemented to produce single feature sets:

(A) mean feature values across all phases,
(B) median feature values across all phases,
(C) feature values from 50% phase,
(D) personalised approach allowing the most stable phase to be selected for each patient.

The most stable phase was defined as the phase with minimum sum of difference in radiomic feature value compared to its two neighbour phases. Initially, for each patient *n*, each feature was considered separately. For each feature value (*X*_*F*_) extracted from each phase (*Ph*) in turn, the sum of the difference in value compared to the two neighbour phases is

\begin{eqnarray*}\begin{array}{c}\begin{array}{lll}{\mathrm{\Delta }}{X}_{F,n,Ph} &amp; = &amp; ABS\left({X}_{F,n,Ph}-{X}_{F,n,Ph-1}\right)\\ &amp; &amp; +\,ABS\left({X}_{F,n,Ph}-{X}_{F,n,Ph+1}\right)\end{array}\end{array}\end{eqnarray*}

calculated in a cyclic way so that when *Ph* is 0%, *Ph* − 1 is 90%, and *Ph* is 10%.

**Figure 2.**
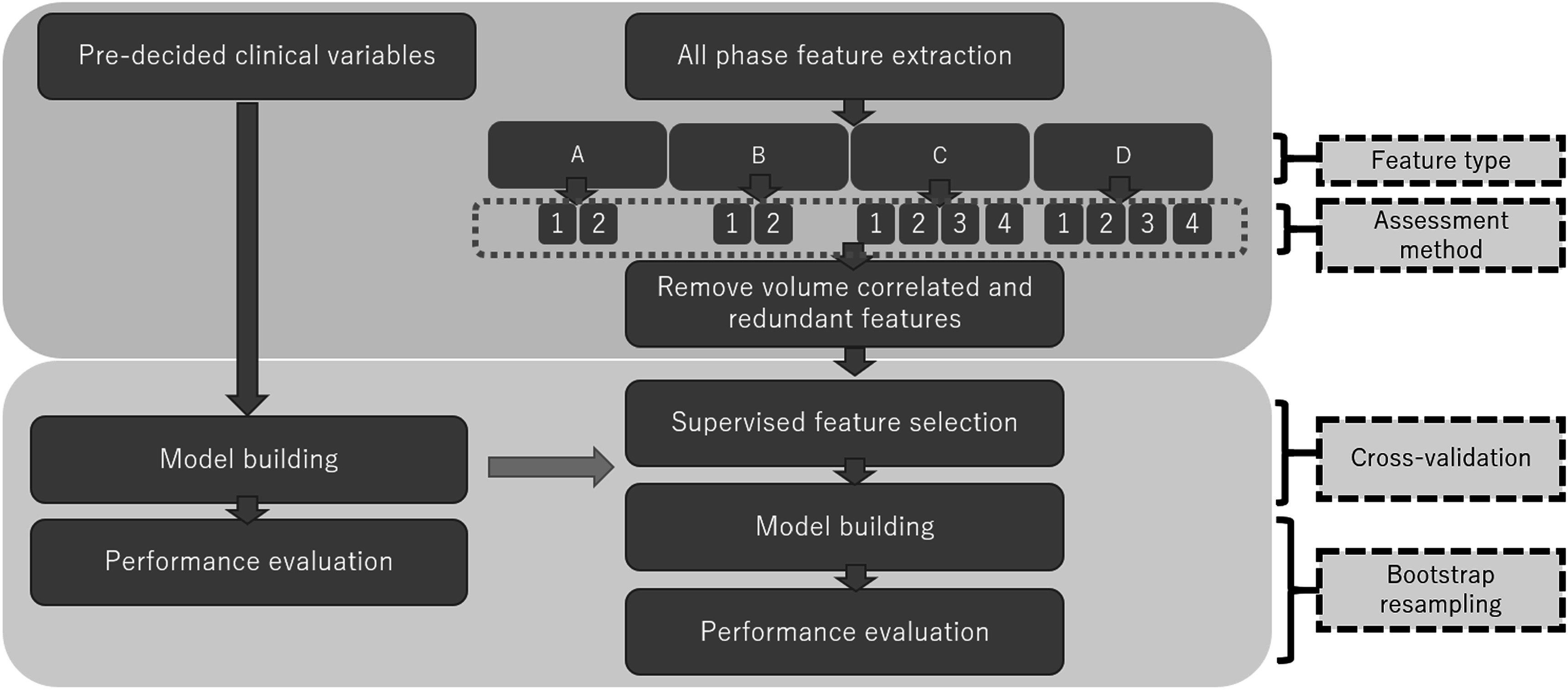
Flow diagram of radiomics analysis. A–D refer to different feature types: mean, median, 50%, and personalised respectively. Boxes 1–4 indicate the different stability assessment methods (all features, unstable features across 10 phases removed, unstable features across neighbouring phases removed, and all features averaged across neighbour phases) implemented on the corresponding feature type. The section highlighted with a yellow background includes an internal validation process (cross-validation and bootstrap resampling).

The phase which results in minimum Δ*X* across comparisons is most stable for each patient-feature pair. The most common phase occurring across all radiomic features for each patient was selected as the most stable phase, and the corresponding feature values were stored for analysis. The final feature sets were used independently in the full analysis process displayed in figure 2.

### 2.6. Stability assessment

To assess whether stability assessment affects prognostic performance we analysed four approaches:

(1) All features included *(None),*
(2) Enforce stability across ten phases *(Stability (*10)),
(3) Enforce stability across neighbour phases *(Stability (*3)),
(4) Averaging across neighbour phases *(Averaging (*3)).

A two-way mixed effects intraclass correlation coefficient was used to assess absolute agreement of feature values across phases compared to a single phase, ICC(A,1)—implemented across all phases for method 2, and three phases for method 3. A lower bound of the 95% confidence interval (CI) greater than or equal to 0.85 was defined as stable, which represents good-to-excellent agreement (Koo and Li 2016), and compares with reported thresholds (Larue *et al* 2017, Tanaka *et al* 2019). The suitability of this threshold was investigated by exploring the relationship between number of stable features for different threshold levels, and influence of tumour motion and volume on stability was considered.

As shown in figure 2, assessment methods 1 and 2 apply to all feature sets (A–D), but, 3 and 4 involve neighbour phases and are only appropriate for single phase cases (C and D). For averaging (method 4), the mean of neighbour phases is stored for analysis (i.e. if 70% is most stable, result is the mean of 60%, 70%, and 80%). Overall, 12 models are created, summarised in table 1.

**Table 1.** The radiomic models studied which are formed from a suitable combination of a method to extract a single feature set from phase feature values and a level of stability assessment.

Models compared
Personalised phase selection
• All features
• Unstable across 10 phases removed
• Unstable across neighbour phases removed
• Average features across neighbour phases
50% phase
• All features
• Unstable across 10 phases removed
• Unstable across neighbour phases removed
• Average features across neighbour phases
Mean across phases
• All features
• Unstable across 10 phases removed
Median across phases
• All features
• Unstable across 10 phases removed

### 2.7. Feature selection and model building

#### 2.7.1. Unsupervised feature selection

For each feature type-assessment combination, correlation with tumour volume was investigated with Spearman rank correlation coefficient (*ρ*), and features with *ρ* above 0.5 were removed. Next, TFs and PFs were investigated independently for redundancy (linear correlation to other features) using Pearson correlation coefficient. A threshold of greater than 0.5 in correlation coefficient highlighted correlated pairs, and the feature in the correlated pair with the largest average correlation to all other features was removed. All features were standardised to mean zero and unit variance, performed prior to cross-validation as the selected feature selection techniques are not influenced by normalisation.

#### 2.7.2. Supervised feature selection

Three supervised feature selection algorithms were tested independently. The methods implemented selected features that: (1) are significantly associated with outcome in a univariable Cox regression (*p* < 0.05), (2) significantly improve a multivariable Cox regression of clinical variables in a likelihood-ratio (LR) test (*p* < 0.05), and (3) have a positive contribution based on minimum redundancy maximum relevance (MRMR) ranking (De Jay *et al* 2013). Each technique was implemented independently over 200 samples created from 40 five-fold stratified cross-validation (SCV) with event-matching.

In each training run, selected features are combined with clinical variables to form a clinical-radiomics Cox model, which was applied without change to the test data. The concordance index (c-index) was calculated for both training and test models with the median and 95% CI across SCV runs recorded.

The feature selection technique that maximised performance whilst maintaining a balance between training and test data was selected. To implement, we calculated a performance ranking from the median c-index across all clinical-radiomic models for both the training (${C}_{train}$) and test (${C}_{test}$) data

\begin{eqnarray*}\begin{array}{c}Performance\,ranking={C}_{test}-\left|{C}_{test}-\,{C}_{train}\right|\end{array}\end{eqnarray*}

which is often used for hyper-parametrisation (Rabasco Meneghetti *et al* 2021).

For the chosen method, the selected features from each training run were recorded and ranked by occurrence. The radiomics signature for each feature type-assessment combination was formed from the top ranked features, with the number of features determined by the median signature size across all SCV runs.

#### 2.7.3. Model building

Based on cross-validation results, a clinical model and 12 clinical-radiomics models were built using the complete dataset. For each clinical-radiomics model (CR), we calculated the adequacy index of the baseline clinical model (C), and subsequently calculated the fraction of new information provided by the radiomics signature

\begin{eqnarray*}\begin{array}{lll}Fraction\,of\,new\,information &amp; = &amp; 1-Adequacy\,index\\ &amp; = &amp; 1-\left(\displaystyle \frac{L{R}_{C}}{L{R}_{CR}}\right),\end{array}\end{eqnarray*}

where LR is the likelihood ratio test ${\chi }^{2}.$ This model performance comparison metric is recommended by Harrell, as difference in c-index (although common for radiomics studies Lambin *et al* 2017) is a low-power metric for interpreting the added value of radiomic features in a clinical model (Harrell 2015). The fraction of new information is the proportion of explainable variation in outcome that is provided by the radiomics signature. The prognostic features were studied for interpretation.

For comparison to radiomics literature, we also calculated the median and 95% CI of the c-index for each model across 500 bootstrap resamples. Following Steyerberg (2009) and Harrell (2015), each bootstrap model was fit to the original data without change and c-index was calculated. Statistical analysis was performed in R version 4.0.2.

## 3. Results

### 3.1. Patient eligibility

Six patients were excluded prior to ROI segmentation: three had at least one phase missing, two had contours missing, and one had multiple lesions at treatment. Seven patients were excluded due to poor registration on visual assessment, and two for not meeting the volume threshold. Median follow-up time for all 258 remaining patients was 18 months (95% CI 15–20 months), and 44 patients experienced distant failure.

The patient demographics are shown in table 2, with the level of missing data reported. Histological subtype data was limited, many patients were diagnosed radiologically as poor health precluded a biopsy. Assessment of comorbidity score was limited due to incomplete reporting in the electronic patient record. Consequently, comorbidity score and histological subtype were not included in the multivariable analysis, as the exclusion was not thought to impact comparison of radiomic models. In analysis of lobe location, ‘middle’ was combined with ‘upper’ for ease of comparison. ‘ECOG 0’ was also combined with ‘ECOG 1’ due to the sparse data in the lowest performance status group.

**Table 2.** Patient demographics table for 258 patients. Total Number column records the number of patients with complete data for each variable, also expressed as a percentage of the total number of patients in the dataset. Tumour volume represents the generated GTV volume on 50% phase. Missing category in histological subtype includes those with radiological diagnosis. ECOG: Eastern Cooperative Oncology Group. ACE-27: Adult Comorbidity Evaluation 27.

Variable	Categories	Number	Total number
Sex	Male	133	258 (100%)
	Female	125	
Age	Median (range)	76 (45–93)	258 (100%)
T stage	T1	152	230 (89.1%)
	T2	77	
	T3	1	
	*Missing*	*28*	
Performance status (ECOG)	0	3	225 (87.2%)
	1	78	
	2	117	
	3	27	
	*Missing*	*33*	
Tumour volume (cm^3^)	Median (Range)	4.02 (0.31–33.8)	258 (100%)
Comorbidity score (ACE-27)	None	7	193 (74.8%)
	Mild	46	
	Moderate	69	
	Severe	71	
	*Missing*	*65*	
Tumour lobe location	Lower	84	252 (97.7%)
	Middle	13	
	Upper	155	
	*Missing*	*6*	
Histological subtype	Adenocarcinoma, NOS	47	116 (45%)
	Squamous cell carcinoma	42	
	Carcinoma, NOS	18	
	Other	9	
	*Missing*	*142*	
Tumour motion amplitude (mm)	Median (range)	5.48 (0–34.3)	258 (100%)

**Figure 3.**
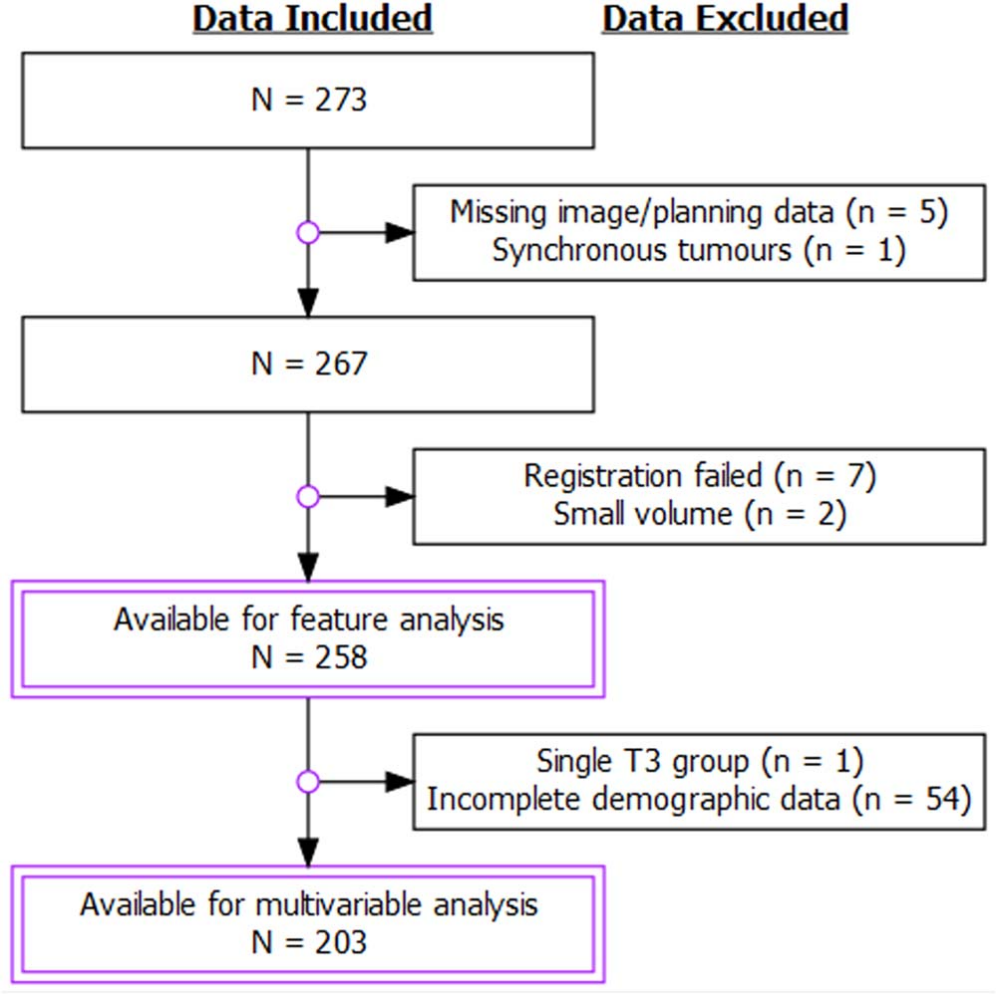
Flow diagram to demonstrate steps implemented to arrive at data available for analysis against outcome. *N* represents the number of patients remaining, whilst n represents those removed.

All stages of data exclusion are shown in figure 3. The 258 patients recorded in table 2 were considered throughout the first stage, however, 203 with complete information were used for feature selection and model-building of which 37 experienced distant failure.

### 3.2. Feature extraction and creation of feature sets

As we did not consider tumour deformation, small changes in tumour volume across phases result from small changes in border voxel inclusion. For 68% of patients the tumour volume was the same across all phases, overall, the mean difference was 0.09 cm^3^ with range 0–1.61 cm^3^. The minimum intensity value differed across patients (SM, section 3).

**Figure 4.**
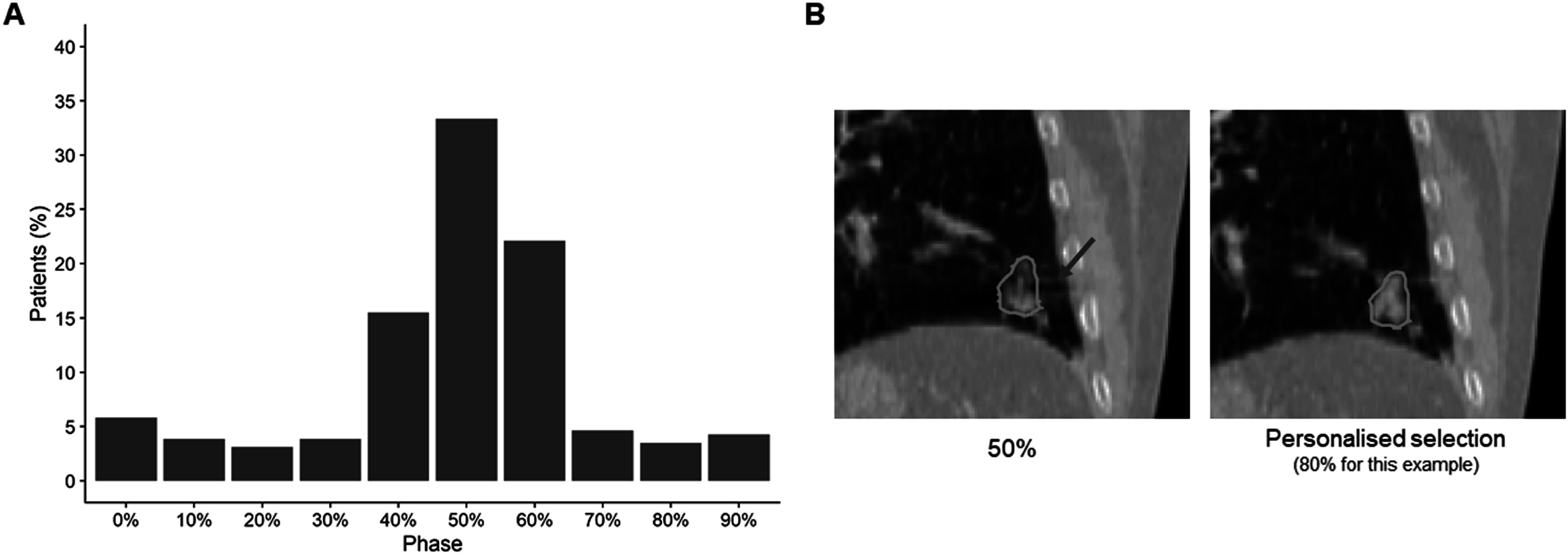
(A) Frequency bar chart of the most stable phase selected for each patient. Exhale phases are most often selected but the full range of phases are chosen. (B) Patient example of 50% versus personalised selection. 50% phase was not suitable for this patient due to presence of a potential artifact (red arrow) which influenced the tumour appearance (tumour outlined with lime contour).

The following feature sets were evaluated: mean, median, 50%, and personalised. The personalised approach selects the most stable phase for each patient individually. For most patients, the most stable phase was close to exhale, with 50% selected for 33%. However, there was not one suitable phase for all patients as the full range of phases were selected (figure 4(A)). Personalised phase selection successfully reduced the chance of using a phase with an artifact in radiomics analysis (figure 4(B)).

Phases were chosen based on majority of features, but, the most stable phase differed per feature, and the selection will depend on what features are used in analysis.

### 3.3. Stability assessment

Exploring all features, 34% of TFs and 40% of PFs were unstable across ten 4D phases, with an overlap of 41 features common across the two ROIs. 11% of TF and PF were unstable across the neighbour phases to 50% and personalised. This classification is largely dependent on chosen ICC threshold, and data studied, as variability is influenced by tumour motion and tumour volume (SM, section 4).

### 3.4. Unsupervised feature selection

The percentage of features remaining at each stage of unsupervised feature selection (stability assessment, volume correlation, and redundancy) are shown in table 3. A similar proportion of features were removed regardless of feature type and assessment method, with less than 6% remaining in all cases. The most features remained after averaging across neighbour phases, and typically for the personalised or 50% phase across each method.

**Table 3.** The percentage of features (*N*%) remaining after each stage of feature selection for the different feature type and assessment combinations. Each column represents a stage of feature selection: stability assessment, volume correlation, and correlation to other features (redundancy). Averaging (3) and Stability (3) refer to assessment methods including the neighbouring phases (i.e. 3 phases in total), and Stability (10) refers to all phase assessment.

	Stage of unsupervised feature selection		
	Stability	Volume	Redundancy
**None**			
Personalised	100	54.8	5.4
50	100	56.2	5.1
Mean	100	53.8	4.8
Median	100	54.0	5.1
**Stability (10)**			
Personalised	62.6	30.4	2.7
50	62.6	31.2	3.0
Mean	62.6	29.0	2.4
Median	62.6	29.3	2.7
**Stability (3)**			
Personalised	89.2	50.5	4.0
50	89.0	52.2	4.8
**Averaging (3)**			
Personalised	100	53.2	5.6
50	100	53.8	5.4

% of features remaining at each stage.			

### 3.5. Supervised feature selection

**Figure 5.**
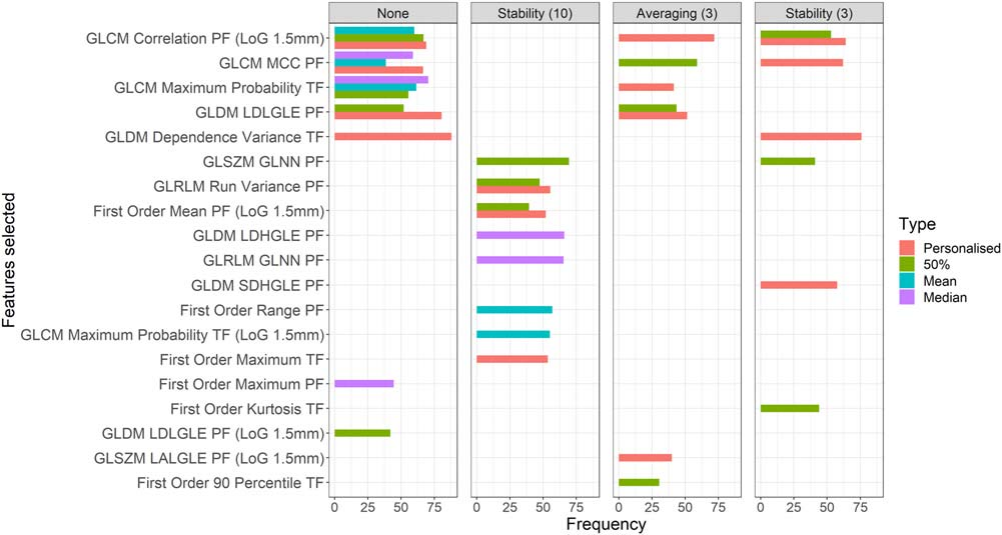
Bar chart to represent frequency of feature selection ranking across cross-validation runs for different feature type (colours), and assessment method (panels) using MRMR feature selection. Frequency represents the percentage of features selected across SCV runs.

Supervised feature selection influenced model performance; however, the top ranking of feature type-assessment combinations was consistent (SM, figure 4). There was typically a larger difference across 4D frameworks than feature selection techniques. The median performance ranking (equation (2)) was 0.42 for univariable, 0.4 for multivariable, and 0.43 and MRMR, and similar signature lengths were derived across techniques (SM, figure 5).

Following implementation of MRMR (highest ranking), the median signature size across 4D frameworks ranged from 2 to 4 features. The median size was used to select the top ranked features, the frequency of occurrence of which is displayed in figure 5. Stability (10) assessment leads to a completely different set of features selected compared to no assessment, however, there are similarities across other techniques.

### 3.6. Prognostic model results

**Figure 6.**
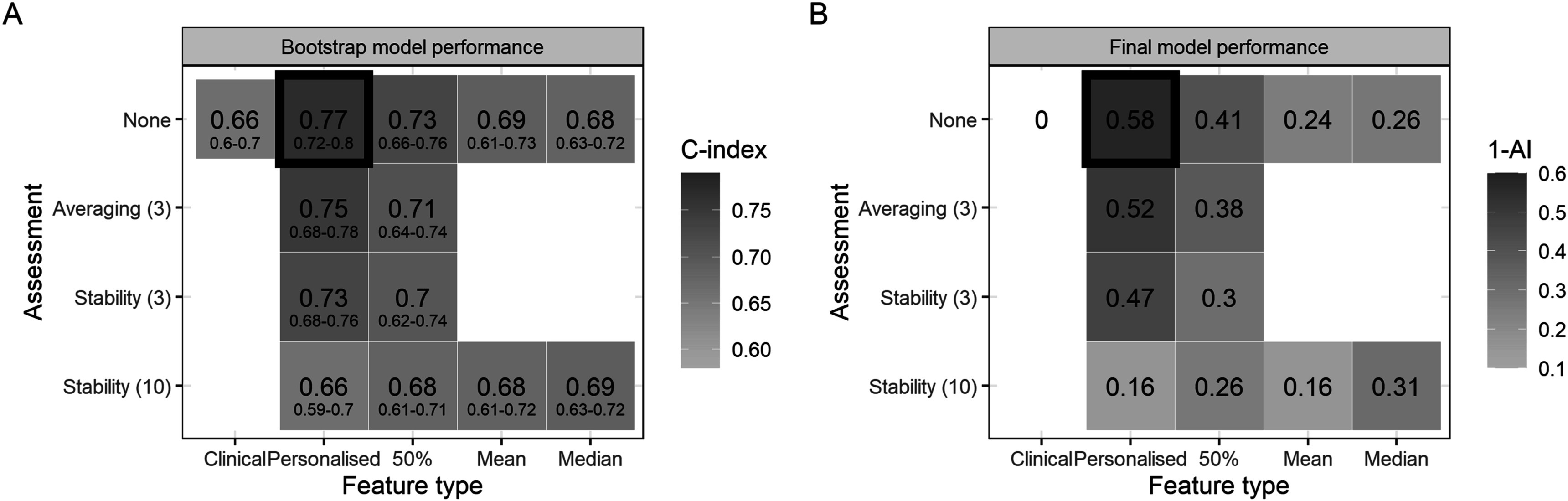
(A) Median and 95% confidence interval of the concordance index (c-index) across 500 bootstrap resamples, and (B) the fraction of new information provided by the radiomics signature compared to the baseline clinical model, which is equivalent to 1 minus the adequacy index (AI). Performance is reported for all 12 models built with different feature set and assessment method, with the clinical model included for reference. The best performing model is highlighted by a black box.

The c-index and fraction of new information for the final clinical-radiomics models are displayed in figure 6. The baseline clinical model is reported in SM, table 3, with tumour volume, tumour lobe location, and biological sex as significant predictors of distant failure.

All radiomic models outperform the clinical model, with radiomic features offering new information to distant failure prediction. The best performing model used the personalised phase with no additional stability assessment. For both no assessment and averaging across neighbouring phases in the personalised case, the radiomics signature has a greater than 0.5 fraction of new information i.e. a greater than 50% proportion in the explainable variation is provided by the radiomics signature. Discarding unstable features over all ten phases has a large negative impact on model performance (<20% new information).

**Figure 7.**
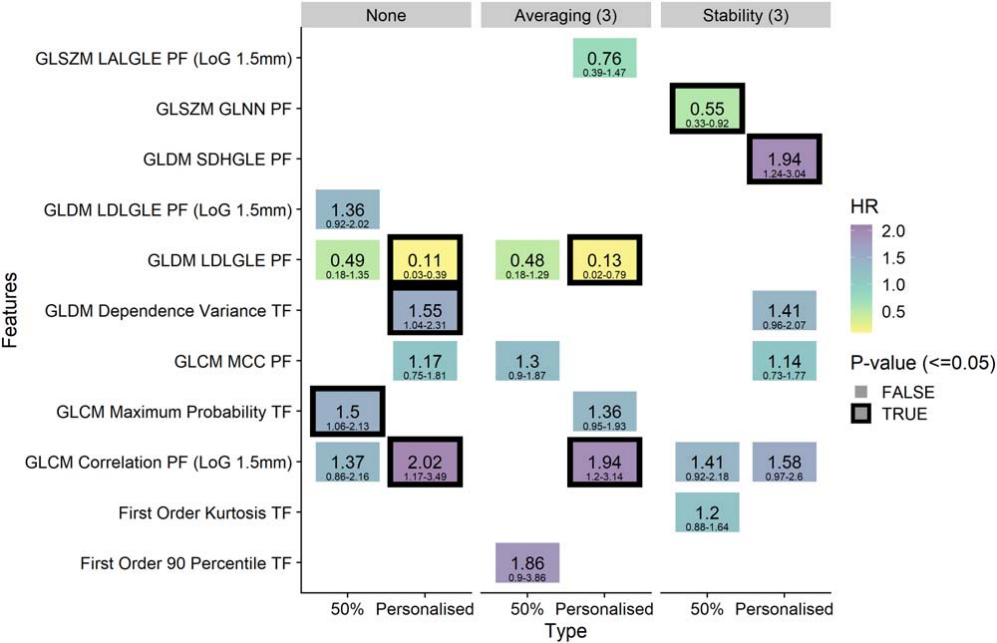
Hazard ratio heat map for the radiomic features included in the models produced by the personalised and 50% phase selection with the assessment methods: none, averaging (3), and stability (3). A significant *p*-value (<0.05) is highlighted by a black box around the relevant feature. The most features are selected in the personalised model.

For each model, different features were included in the radiomics signature that have varying prognostic value. In figure 7, the multivariable hazard ratio and significance for features included in the best performing models are displayed i.e. single-phase features with no assessment, or assessment performed over neighbouring phases. Other results are included in SM, section 7 for completeness.

Across all cases the personalised model contains more features that are significant after adjusting for clinical variables (*p* < 0.05). For no stability assessment, the PFs GLCM Correlation calculated on the LoG image and GLDM large dependence low grey level emphasis (LDLGLE) are included in the 50% and personalised model, but only significant in the personalised case. These features remain significant in the personalised model after averaging is performed, but, GLCM correlation does not remain significant after stability (3) assessment is implemented. For Stability (3), GLDM LDLGLE is replaced with GLDM small dependence high grey level emphasis, which are correlated (*ρ* = −0.52).

TF GLCM maximum probability is statistically significant in the 50%, mean, and median model with no stability assessment (SM, section 7). This feature is highly correlated to the TF GLDM Dependence Variance which is significant in the personalised model (*ρ* = 0.89).

## 4. Discussion

In this study, we have a developed a method to select the most stable 4D-CT phase for radiomics analysis. The method improved model performance for distant failure prediction compared to the 50% phase which is often assumed most stable (Lafata *et al* 2018), or averaging ten phase feature values—which offered worst performance regardless of whether mean or median averaging was used. The most stable phase was selected as 50% in 33% of cases but varied across the whole range. The best model performance was achieved when traditional stability assessment was omitted, this step would remove features requiring sharp image detail that only occurs in one or a few phases. In summary, a single stable 4D phase led to better model performance, likely because it represents small anatomical details with a higher resolution, therefore features sensitive to motion should not be omitted. A data-driven approach to selecting the phase achieves superior results and could aid multi-institutional studies where different 4D protocols are adopted.

In all models, prognostic features for distant failure were found. In the personalised model, TF ‘GLDM dependence variance’ was significant, with an increased risk of distant failure for a higher variance. PF ‘LDLGLE’ of GLDM in the personalised model was prognostic even after averaging across neighbour phases was implemented. This feature detects high dependence groups of low intensity (Sun and Wee 1983), and a higher value leads to a protective effect (HR < 1). This feature was also included in models after stability assessment. After stability assessment across neighbour phases, PF ‘Small Dependence High Grey Level Emphasis’ of GLDM was a significant feature included in the model, stating that small dependence groups of high intensity around the tumour border offer increased risk. With no assessment, TF ‘GLCM maximum probability’ was significant for mean, median, and 50%, a higher probability of the most common GLCM leads to worse prognosis (Haralick *et al* 1973).

More PFs than TFs are included in the clinical-radiomics models suggesting PFs are more important for distant failure prognosis. In summary, higher intensity groups outside the tumour border may lead to worse prognosis. Out of clinical features, tumour lobe location is prognostic, with lower lobe tumours performing worse, potentially related to overperfusion at the lung bases (Shaverdian *et al* 2017). Such conclusions are hypothesis-generating until validation or larger sample size studies are performed. Overall, evidence is building to suggest importance of the peritumoural region for distant metastasis prediction from both pathology and imaging studies (Shimada *et al* 2010, Kadota *et al* 2015, Dou *et al* 2018). Such a biomarker would improve current clinical prediction as patient factors (i.e. stage, ECOG performance status, and age) are not consistently prognostic for distant failure (Loganadane *et al* 2016, Miller *et al* 2019).

To extract imaging biomarkers a single phase is preferable to averaging across all phases, as it can provide useful information for features highly sensitive to respiratory motion (Lafata *et al* 2018). In this work, we establish that requiring stability across phases is highly detrimental in 4D-CT radiomics. This is in contrast to the improved prognostic performance stability assessment has for FB-CT studies where there is large motion blurring impacting feature extraction (Du *et al* 2019). In this study, feature stability was dependent on tumour motion (as also found by Tanaka *et al* 2019), and tumour volume. Alternative measures to stability assessment could be tested such as identifying features sensitive to small shifts between the image and ROI mask to avoid segmentation ‘style’ causing bias (Zwanenburg *et al* 2019a), and use of test-retest feature selection. To provide a potential balance between robustness and model performance we suggest to average phases around the phase selected or only require stability across three phases, this reduced model performance but was still preferable to the 50% phase or averaging across all phases.

The most stable phase for each patient was most often near exhale (40%–60%), followed by inhale peak (0%). This is expected as mid-exhalation phases are typically the least stable; the tumour moves fastest during these phases resulting in the largest shape deviations compared to FB-CT (Rietzel *et al* 2006). Interestingly, mid-phases were selected for a small proportion of patients, further supporting a data-driven approach for phase selection as opposed to an assumption across the cohort. One possible explanation for this behaviour could be cardiac motion that is uncorrelated with respiratory motion and can be minimal at any phase i.e. phase with the least motion is not always exhale. Another explanation could be the phase-binning approach implemented, in this study 50% is not always the true end-exhale unless patients have sinusoidal breathing. Alternatively, artifacts occur in over 90% of 4D-CT images (Yamamoto *et al* 2008), and presence of artifacts reduce suitability of end-exhale. All explanations make a data-driven approach favourable, to select the best method to account for 4D protocol, patients’ respiratory differences, and an individual tumours proximity to the heart.

Each 4D phase uses less imaging dose in comparison to composite and FB-CT scans. A single phase may therefore display more heterogeneity than composite scans due to increased noise. Composite scans reduce noise but also limit the visibility of small anatomical details. AIP features have been studied for distant failure prediction (Huynh *et al* 2017), but results are inconclusive (Lafata *et al* 2018). We did not study AIP as data is blurred due to tumour motion. An alternative is the motion compensated (mid-position) scan (Wolthaus *et al* 2008), which displays the tumour at an average position with reduced noise as multiple phases are combined. This seems favourable, but does require assessment in future work, as limitations in deformable registration accuracy could still affect subtle details (Mercieca *et al* 2018).

Equally as important for future consideration, an automated method was used to generate a GTV on every phase, allowing for large-scale analysis without the need for manual contouring. This methodology could introduce small errors in the shape of the tumour, but it is validated to work well within the limits of observer variation and improve prognostic modelling (Davey *et al* 2020a). However, the tumour border largely influences radiomic features (Pavic *et al* 2018), and a potential limitation is that only a single segmentation technique was tested. If the model was intended for clinical purposes, multiple segmentations are required to obtain a better radiomics quality score (Lambin *et al* 2017), and to incorporate uncertainty in modelling or as a feature selection stage. Additional considerations for a clinical study would include resampling methods and bin discretisation. Such tests have been omitted in this study to focus on the sole issue of optimising phase selection for 4D-CT imaging biomarker studies. The segmentation method also assumes deformation of the tumour is small compared to translational motion. Larue *et al* allowed for tumour deformation when comparing 4D phases and found 32% of tumour shape features are unstable across all phases (Larue *et al* 2017), however, this only measures the opinion of one observer with manual delineation on every phase. This is not feasible in larger cohort studies, and without manual assessment it is difficult to distinguish between biological shape changes, and that due to artifacts. Biomechnical models that account for tumour deformation have been developed and tested in small cohorts (Jafari *et al* 2021), and could aid extension of the segmentation methodology in future.

Another limitation of traditional radiomics is that spatial information is collapsed to single feature values to describe a region-of-interest. To incorporate more information we tested two ROIs: the tumour and the peritumour arbitrarily defined based on work by Dou *et al* (2018). The proposed border may not be optimum for predicting distant failure, but the generated hypothesis can be used to investigate this further with novel techniques that maintain spatial information (Davey *et al* 2020b). Well known to the radiomics community, the ROI volume strongly influences feature values and is a major confounder. In this study we removed features with correlation coefficient of greater than 0.5 with tumour volume, this is an arbitrary threshold but correlation assessment is common with often more lenient thresholds implemented (Li *et al* 2017). In previous work, we have noted even weak correlations can have an impact on the apparent prognostic nature of radiomic features, so potentially significant features would have to be explored further to assess clinical relevance (Davey *et al* 2020c). Of course, with any feature selection approach clinically relevant parameters can be lost (Leger *et al* 2017). Furthermore, small tumour volumes may not be adequately sampled to extract all features. We ensured all ROIs were above the size limit of 64 voxels (as implemented by LifeX, Nioche *et al* 2018), but this limit has not been formally evidenced and further research is required to determine what threshold is suitable for different features.

A main limitation of our study is the sample size for analysis, but, this remains one of the larger sample size SABR radiomic studies, as typically no more than 170 patients are studied (Huynh *et al* 2017, Lafata *et al* 2019, Li *et al* 2017, Oikonomou *et al* 2018). In addition, the internal validation implemented increases confidence in the conclusions. To further increase confidence, we tested three supervised feature selection techniques following advice of Leger *et al* (2017). After feature selection, we implemented a simple Cox regression model. This has been shown to perform just as well as more complex models (i.e. boosted-Cox), and feature selection is the area which is more prone to error (Parmar *et al* 2015a, 2015b, Leger *et al* 2017). We selected the feature selection method that had the best performance in cross-validation whilst minimising difference between train and test, which was the MRMR assessment. Alternative methods to comparing multiple feature selection methods exist: such as assessing the stability (Parmar *et al* 2015a), or creating an ensemble model. Regardless of technique implemented the conclusions remained the same, with optimal performance of the personalised approach.

As the gold-standard breath-hold CT is not standard of care, we have suggested a method to obtain the single phase of optimal quality for biomarker assessment (Scrivener *et al* 2016, Oliver *et al* 2017). The personalised approach of selecting the most stable phase will allow for prognostic information to be identified which is lost with current approaches of either requiring stability assessment across all phases, or assuming the 50% phase (exhale) is optimal. Although this framework has been evaluated in the context of traditional radiomics where many features are tested, it is also useful to consider when testing a single imaging biomarker on 4D-CT. In this study, we used all radiomic features to inform selection of optimal phase. To extend to a single biomarker study, two approaches could be tested: selecting the optimal phase for the single feature or using motion-sensitive comparison scores (e.g. difference in radiomic features) to determine the most stable phase.

## 5. Conclusion

Assessment of feature motion stability is important for radiomic analysis on FB-CT data, but reduces model performance and removes prognostic features from the final model when using a single phase from a 4D dataset. To increase model performance, extracting features from a single phase is preferable to averaging across all phases. To select the single phase adopting our personalised approach is superior to assuming the 50% phase is optimal for all patients. This remains the case when averaging the most stable and neighbour phase features to reduce noise. Overall, we have set up a framework to perform radiomics analysis on 4D-CT and highlighted a clinical hypothesis to take forward for validation and further study.

## Sources of support

This was supported by CRUK via the funding to Cancer Research Manchester Centre [C147/A25254]. Professor Marcel van Herk, and Professor Corinne Faivre-Finn are supported by NIHR Manchester Biomedical Research Centre.
